# Neurosensory Prosthetics: An Integral Neuromodulation Part of Bioelectronic Device

**DOI:** 10.3389/fnins.2021.671767

**Published:** 2021-11-16

**Authors:** Ifeoma Ezeokafor, Archana Upadhya, Saritha Shetty

**Affiliations:** Shobhaben Pratapbhai Patel School of Pharmacy & Technology Management, Shri Vile Parle Kelavani Mandal (SVKM) Narsee Monjee Institute of Management Studies (NMiMS) (SVKM’S NMiMS), Mumbai, India

**Keywords:** bioelectronic devices, bioelectronic medicine, neuromodulation, vagus nerve stimulation, retinal implants, auditory implants

## Abstract

Bioelectronic medicines (BEMs) constitute a branch of bioelectronic devices (BEDs), which are a class of therapeutics that combine neuroscience with molecular biology, immunology, and engineering technologies. Thus, BEMs are the culmination of thought processes of scientists of varied fields and herald a new era in the treatment of chronic diseases. BEMs work on the principle of neuromodulation of nerve stimulation. Examples of BEMs based on neuromodulation are those that modify neural circuits through deep brain stimulation, vagal nerve stimulation, spinal nerve stimulation, and retinal and auditory implants. BEDs may also serve as diagnostic tools by mimicking human sensory systems. Two examples of *in vitro* BEDs used as diagnostic agents in biomedical applications based on *in vivo* neurosensory circuits are the bioelectronic nose and bioelectronic tongue. The review discusses the ever-growing application of BEDs to a wide variety of health conditions and practices to improve the quality of life.

## Introduction

Bioelectronic devices (BEDs) combine a biosensing material, such as a protein, nucleic acid, and cells, with electronic circuitry. Bioelectronic medicines (BEMs), on the other hand, are a separate class of BEDs where electronics are used to manipulate electrical signals *in vivo*. BEMs can be classified into various categories based on the type of electrode employed (geometry/size/material of manufacture), location of implantation (retina, ear, brain, spinal cord, and peripheral nerve), type and strength of stimulation (in terms of frequency of charge, pulse, and pulse interval), and type of system (open loop, closed loop, and smart).

Bioelectronic medicines find wide applications in many disease conditions caused by or leading to dormant or aberrant neural networks. In BEMs, the electric signal-modifying element (the electrode) is placed *in vivo* at a specific location and is manipulated externally through a processing unit. This artificial modulation of electric impulses transmitted through neurons is an upcoming branch of science termed as neuromodulation. The International Neuromodulation Society defines neuromodulation as the alteration of nerve activity through targeted delivery of a stimulus, electrical or chemical, to specific neurological sites in the body (De Ridder et al., 2021).

Neurons are sensitive to stimulations at synaptic junctions that cause them to depolarize, and the wave of depolarization is extended through their axons to other synaptic junctions that culminate in the brain or other organs. The message is, thus, transmitted to central regions that regulate the homeostasis of an individual. Electrical, magnetic, optogenetic, thermal, acoustic/mechanical, and chemical stimulations can alter the activity of neuron (Luan et al., 2014). Prior to the application of a given neuromodulation technique to a tissue, its safety, efficacy, and suitability (Luan et al., 2014) must be considered.

Retinal and auditory BEMs convert optic and acoustic stimulations, respectively, to electrical signals that are then perceived by the brain. Other BEMs discussed in the review rely on the external modulation of electrical impulses to ameliorate neurodegenerative disorders, improve cardiovascular functions, reduce inflammatory conditions, and reduce chronic pain experiences. The knowledge of neurosensory circuitry can be employed in therapy and can be used to prepare *in vitro* sensory devices that can mimic human functions, thus decreasing the need of human exposure for validation of certain tests. The bioelectronic tongue and nose discussed here are examples of such devices.

## Implanted Electrodes as a Bioelectronic Medicine Component

A BEM consists of a neural interface that has the function of stimulating a population of neurons or nerve fascicles and recording their electrical output, to enable their manipulation in order to restore the physiological neural activity of the tissue (Redolfi Riva and Micera, 2021). The BEM containing conductive elements is geometrically designed with special features that enable it to interact with the tissue of interest (cortical, spinal, or peripheral; Redolfi Riva and Micera, 2021). The chapter by Tyler (2018) is recommended to readers who wish to learn more on the intricate design of electrodes with respect to the implanted tissue and many other factors. An ideal BEM implant should provide stable electrical performance, and should be biocompatible with the tissue of implantation to prevent a host-induced inflammatory reaction. Host-induced inflammation, called “foreign body reaction” (FBR), is a natural protective mechanism of the host body that alters the function of the implanted device (Zhang et al., 2020). Injury caused during implantation triggers wound healing and immune cell signaling cascades that subsequently lead to fibrosis and collagen encapsulation of the implant. Formation of the proteinaceous capsule around the device increases its resistance to electrical conduction, thus compromising its intended effects (Tyler, 2018; Redolfi Riva and Micera, 2021).

Briefly, in initial phases of FBR, protein gets attached/layered on the implant recruiting immune cells at the site, which are activated depending on the type and amount of adsorbed protein. The activation of immune cells triggers complex responses at the site, such as release of proteolytic enzymes, reactive oxygen species, and pro-inflammatory mediators, leading to inflammation (Zhang et al., 2020). Acute inflammation is followed by chronic inflammation, which later subsides, resulting in a dense collagenous encapsulation of the implant, isolating it from the host body, and causing a subsequent loss of function. Neuro-prosthetics, are particularly vulnerable to FBR, since the fibrotic encapsulation damages and displaces the target neuronal tissue, and forms a high-impedance layer that weakens the interception of electrode signals and dissipates stimulating electrode currents (Carnicer-Lombarte et al., 2021).

Strategies that have been employed to mitigate FBR include modification of physical properties of a device, delivery of anti-inflammatory agents to suppress inflammatory responses, and modification of implant surface with bioactive agents/biomaterials (Zhang et al., 2020).

Physical properties of the device include the size, shape, roughness, surface topography, and mechanical strength of materials used (Zhang et al., 2020), and these may be suitably adjusted (Mariani et al., 2019). Types of materials used in the fabrication of tissue electrodes previously were metal wires, such as tungsten, platinum, platinum/iridium, iridium, and titanium, and non-metals such as silicon. These, however, have a limited life span in the body due to FBR and material instability (Hejazi et al., 2021). To overcome the limitation of an inflammatory tissue response, flexible polymeric materials, such as polyimide (PI), Parylene-C, and SU-8, may be employed, which integrate well in neural tissue and remain active for months (Hejazi et al., 2021). The polymers PI and Parylene-C (p-xylylene) are also explored as insulation substrates to protect electric components from coming in direct contact with body fluids (Wu and Peng, 2019). Polyaniline (PAN), poly(3,4-ethylenedioxythiophene), and polypyrrole (PPy) are other polymers widely used as electrodes (Wu and Peng, 2019). Carbon-based electrodes, such as carbon fibers, carbon nanotubes, and graphene, are being developed since they can be fabricated into low micron dimensions and possess softer surfaces and adjustable electro-chemical properties (Hejazi et al., 2021), which may be suitable for evading FBR or reducing its severity.

To increase electrode compatibility and reduce tissue inflammation, electrodes may be coated by some materials categorized as anti-inflammatory (e.g., dexamethasone, minocycline, alpha melanocyte stimulating hormone, and interleukin -I receptor antagonist), and neuron-promoting (e.g., nerve growth factor, laminin, and Matrigel) (Woods et al., 2020). Biomaterials, such as silk, collagen, protein, polysaccharides (chitin, cellulose), are also being investigated as support materials that may be used in conjunction with the electrode-forming material for better functioning of the BEM (Pradhan et al., 2020).

Approaches to modify an electrode-neural interface to reduce FBR are beyond the scope of this review and are well illustrated in other reviews (Lotti et al., 2017; Carnicer-Lombarte et al., 2021).

## Neuromodulating Devices

### Retinal Implants

**FIGURE 1:**
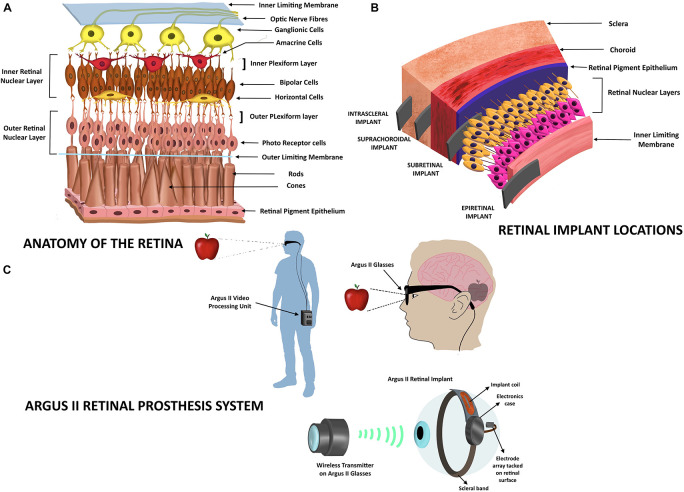
Retinal implants. **(A)** Anatomy of the retinal layer. The figure depicts the organization of the retinal neural layer. The outer nuclear layer comprises nuclei of photoreceptor cells, while the inner nuclear layer consists of the nuclei of the Müllerian glia (not shown here), the bipolar cells, the amacrine and the horizontal cells. The photoreceptor cells synapse with the bipolar cells and horizontal cells. The bipolar and amacrine cells in turn synapse with the ganglionic cells whose axons form the optic nerve fiber. **(B)** Retinal implant locations. The types of retinal implants are represented according to their location in the eye: retinal, subretinal, suprachoroidal, and intrascleral. **(C)** Depiction of the Argus II Retinal Prosthesis system. The video/image (here shown as an apple) is captured by a camera on the glasses and is sent into an external portable processing unit that converts visual data received into electrical data. These data are sent back to the glasses that also contain a transmitter that wirelessly transmits the electrical message to the electrode array that is part of the retinal implant. The electrodes transform the information into electric impulses, which stimulate the retina and bring about transmission *via* the optic nerve to the visual cortex of the brain that perceives the image.

The eye receives light energy, which is transformed to action potentials that travel through the optic nerve and are carried to specific sites in the brain. The sense of vision involves components of the eye (lens, retina, and optic nerve), optic chiasma, the optic tract, the lateral geniculate nuclei, and the geniculocalcarine tract that projects into the occipital cortex of the brain where the images are perceived. The eye is similar to a camera that focuses the light that is refracted by the lens, vitreous humor, and aqueous humor to the retina (the focal center of the eye) (Sánchez López De Nava et al., 2021). The retinal layer is a thin layer of transparent cells, located at the posterior section of the eye, and is separated from the choroid by the retinal pigment epithelium (RPE). Retina is composed of six types of cells: photoreceptor cells, bipolar cells, horizontal cells, amacrine cells, ganglion cells (involved in the reception and processing of visual signals), and Müllerian glial cells (which radially stabilize the retina) (Lin et al., 2015). The retinal layer exhibits cells arranged in parallel configurations (Figure 1A). The nuclei of photoreceptor cells comprise the retinal outer nuclear layer, while the nuclei of the Müllerian glia, the bipolar cells, the amacrine and the horizontal cells form the inner nuclear layer. The synaptic regions of the retina form the plexiform layers. The retina contains two plexiform layers; in the outer plexiform layer the photoreceptor cells connect with bipolar and horizontal cells, and in the inner plexiform layer bipolar and amacrine cells synapse with ganglion cells. The nuclei of the ganglions form the ganglion layer and their axons bundle up to form the optic nerve fiber (Willoughby et al., 2010). The photoreceptor cells transform the received photon light energy into graded neural signals, which are transmitted and processed *via* the bipolar and ganglion cell layers. The optic nerve that emerges from the axons of ganglion layer transmits the images to the visual center in the brain where the images are perceived (Fitzpatrick, 2015).

Retinal implants are used to improve vision in people with degenerative retinal diseases, such as retinitis pigmentosa (RP) and age-related macular degeneration. These diseases cause the deterioration of the outer nuclear layer of retinal photoreceptor cells, while the inner nuclear layer, optic nerve, and other processes that lead to the visual center of the brain are intact. Since the cells of inner nuclear layer transmit signals to the optic nerve, they can be electrically stimulated *via* retinal implants to evoke the downstream visual pathway (Fitzpatrick, 2015; Lin et al., 2015). Retinal implants will not aid the vision of patients who have been blind from birth, since in these patients the visual processing centers are under-developed (Fitzpatrick, 2015). The stimulation strategies of the inner nuclear layer that are being investigated are bioelectronic, optogenetic, photochemical, and ultrasonic stimulations (Yue et al., 2016; Shim et al., 2020). Current retinal prosthetics are categorized based on the location of implant; epiretinal, subretinal, suprachoroidal, or intrascleral (Figure 1B). Epiretinal implants are inserted on the retinal surface with electrodes extending to inner nuclear layers to stimulate bipolar or ganglion cells. Subretinal implants are inserted inside the retina in the photoreceptor layer, while suprachoroidal implants are located at the back of the eye between the retina and the sclera of eye (Fitzpatrick, 2015). These electronic retinal prosthetics only improve visual acuity from blindness to low vision; they do not restore full visual acuity, because the high density of photoreceptors in the fovea (the retinal region accrued with the highest visual acuity) cannot be completely replaced by current microelectronic devices (Lin et al., 2015). In patients implanted with retinal prostheses, images can be perceived as light patterns of monochromatic dots or “phosphenes” (Ayton et al., 2020). The clarity/quality of images visualized by the patient depend on several factors, such as the number of electrodes/photodiodes on the implant, the type of stimulation, and the shades of gray that can be identified by the patient (since images are seen as gray objects; Ayton et al., 2020).

In the epiretinal implant system, the retinal stimulator is placed on the retinal surface (on top of the ganglion layer). The image to be perceived is captured by an extraocular camera, processed by a retinal encoder and the information of the signal pattern is transmitted into the eye through wireless routes (Walter, 2005). For example, The *Argus II system* (Ahuja and Behrend, 2013) consists of a pair of glasses equipped with an internal video camera that sends images to an external battery operated video processing unit (Figure 1C). The processed visual information is sent back to the glasses from where it is transmitted wirelessly to a receiver attached to a scleral band, which encircles the eyeball. The receiver then sends electrical pulses *via* a flat wire through a small incision in the eye to an electrode array that contacts the inner retinal ganglion layer in the retina (Fitzpatrick, 2015). The *Argus II* (Second Sight Medical Products Inc., Sylmar, CA, United States) comprises a 60-electrode array system (6 × 10 round platinum-coated electrodes, 200 μm in diameter, center to center spaced at ∼575 μm) embedded in a thin film of polyimide (Luo and Da Cruz, 2016) and is the only device with approval for treating RP in Europe (2011) and the United States (2013; Trinh et al., 2020). Implantation of this device requires a trained vitreoretinal surgeon to perform an operation called “3-port pars plana vitrectomy” (Ayton et al., 2020). Patients implanted with *Argus II* may be able to read exceptionally large letters (20/1260; Humayun et al., 2012) and locate people or objects. Since *Argus II* has a limited number of electrodes or pixels (∼60), the resolution of the image formed in the brain is compromised (Im and Kim, 2020). The resolution of the normal human eye is many megapixels due to millions of rods and cones in the retina. A healthy human eye contains an average of 4.08 million cones and 92 million rods (Curcio et al., 1990), which transform light energy into electrical impulses, which are then carried forth. *Argus II* wearers are recommended to avoid large eye movements to prevent image displacement. Instead, they are trained to maintain a steady gaze and scan the environment by head scanning (Stronks and Dagnelie, 2014), so that the images could be captured adequately by the external camera and relayed. In the subsequent years after approval, *Argus II* has been implanted in over 350 patients globally. However, in May 2019, the *Argus II* manufacturers announced that they were discontinuing the product. Other epiretinal prostheses under investigation in Europe are the *EPI-RET 3* (Univ of Aachen; Roessler et al., 2009), *IRIS II* (Pixium Vision, France; Muqit et al., 2019) and the *Intelligent Medical Implants* (IMI; Hornig et al., 2007; Lin et al., 2015; Ayton et al., 2020). The risks of epiretinal implantation are poor implant fixation, retinal re-organization due to the implant, and retinal detachment (Villalobos et al., 2013).

In the subretinal approach, the retinal stimulator is placed in the retina. This implant is close to the target retinal layer and benefits from natural retinal signal amplification that requires lower stimulation intensities (Bloch et al., 2019). The surgical implantation of this stimulator requires similar procedures as that of epiretinal implants but is more challenging since there are issues of retina-retinal pigment epithelium adhesion(RPE) due to the underlying degeneration (Bloch et al., 2019). The stimulator consists of hundreds of miniaturized photodiodes (positioned in the layer of photoreceptor cells) that transform light into electrical power and stimulate the inner nuclear layer of the retina. An image processing external unit or camera is not required (Walter, 2005). For example, *The Alpha IMS* (Retinal Implant AG, Reutlingen, Germany) uses an array of photosensitive electrodes that directly stimulate the middle retinal layer and retinal ganglion cells. This implanted device contains 1,500 photodiodes (38 × 40 ∼1,500 square-shaped iridium electrodes, 50 μm × 50 μm, chip dimensions 3.2 mm × 3.1 mm) on a polyimide foil (Stronks and Dagnelie, 2014; Stingl et al., 2015). The power supply required for operation and stimulation current are provided by the receiver coil (placed in a sub-dermal box behind the ear) that is connected to the sub-retinal chip/implant *via trans*-scleral cables (Stingl et al., 2015). The reference electrode placed in the temple or retro-auricular region is connected to the receiver coil by a separate short cable. A transmitter coil (placed above the receiver coil) is managed by a handheld unit, which enables the user to switch on or switch off the device and adjust the contrast sensitivity and brightness (Stingl et al., 2015). *Alpha IMS* was designed to achieve a Landolt C-ring visual acuity of 20/250, but the acuity assessed in 4 of 29 blind patients on implantation was 20/2000, 20/606, and 20/546 (NCT01024803; Stingl et al., 2015). Other parameters measured were light perception (observed in 86% of patients), light source localization (59%), clock task (17%), reading letters (14%), motion perception (21%), and grating acuity (52%; Stingl et al., 2015). Thirteen participants out of the 29 reported improved daily life experiences with the implant, as they could see shapes/details of objects in gray scales. An improved version of this implant, the *Alpha AMS* with 100 additional photodiodes, i.e., 1,600 electrodes (Trinh et al., 2020) underwent clinical trials (NCT01024803, NCT02720640) to test its efficacy in 15 patients who were blind in one eye (Stingl et al., 2017). The *Alpha AMS* subretinal implant works on the same principle as the *Alpha IMS* implant but differs in the following aspects: chip supplier, stimulation pulse/type, electrode shape/dimension, electrode number, chip size, foil substrate, and silicone cable (Stingl et al., 2017). The visual acuity with the device was observed to be 20/546 and 20/1111 in two patients, while light perception was seen in 13 of the 15 patients. The implants depend on an intraocular light sensing system, so normal eye movements may be used (in contrast to epiretinal implants). However, there is limited control over stimulation parameters (Stronks and Dagnelie, 2014). Both these implants received CE (European Conformity) approval but were discontinued with the dissolution of the company in March 2019, although investigations are underway to improve *Alpha AMS* (Ayton et al., 2020). Other subretinal implants under investigation are the *PRIMA* implant (Pixium Vision, France), *Boston Retinal Implant* (Boston Retinal Implant Project) and the *Artificial Silicon Retina (ASR)* from Optobionics. *ASR* contains ∼5,000 independently functioning micro-electrode-tipped micro-photodiodes (electrodes are 20 μm in diameter with 9-μm diameter iridium-tipped microelectrodes, separated by 5 μm) powered solely by incident light with no external power supplies (Chow et al., 2004; Fitzpatrick, 2015; Bloch et al., 2019). In *PRIMA*, photovoltaic pixels (378 in number, 100 μm in diameter, chip 2 mm width 30 μm thickness), are stimulated by near infrared (880 nm) light from external video glasses that process images. A photovoltaic array then converts the pulsed light obtained into pulsed electric current to stimulate the inner nuclear layer of the retina (Palanker et al., 2020). Subretinal implants are associated with risks such as loss of residual photoreceptor layer, re-organization of inner retina, disruption of the RPE, and surgical trauma causing retinal perforations (Villalobos et al., 2013).

Retinal prostheses may also be positioned in the suprachoroidal space. The surgical procedure to place these implants is less invasive compared with that of the epiretinal and subretinal implants. However, the choroidal layer is highly vascular, posing a threat of retinal bleeding, and there is a potential risk of fibrosis post implantation (Bloch et al., 2019). The implant is situated at a larger distance from the neurosensory retina than epiretinal implants posing challenges with respect to retinal stimulation for image perception. The overall greater distribution of current may also lead to a compromise in the image resolution (Bloch et al., 2019). Preclinical studies with the implants [array of 21 platinum (Pt) electrodes fabricated in a 19 mm × 8 mm silicone substrate with 2 Pt return electrodes] on rats were initially performed by Bionic Vision Australia (BVA; Villalobos et al., 2013). Following the study on rats, BVA carried out human trials (NCT01603576) with improved prosthesis implanted in the suprachoroidal space (Ayton et al., 2014) while in human trials conducted by Osaka and colleagues (Japan) the prosthesis was implanted in the scleral pocket (Fujikado et al., 2016), respectively. BVA is now carrying out improvisations in the implant to address the issue of retinotopic discrimination and high stimulation thresholds by designing a 44-channel fully implantable device and a 99-channel device (Phoenix; Bloch et al., 2019).

Apart from surgical procedures required for device implantation, the design of any retinal prosthesis must consider the engineering aspects related to electrode material, number of arrays, packaging of the electrodes into a biocompatible implant, retinal stimulation strategies, and image processing hardware or software (Ayton et al., 2020).

### Auditory Implants

**FIGURE 2:**
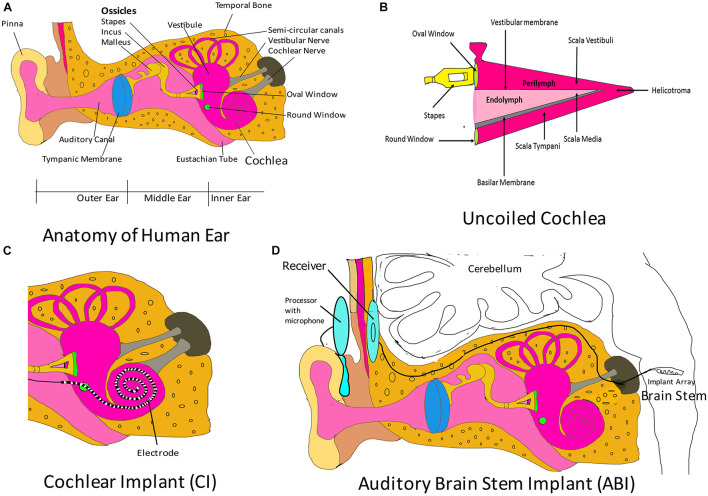
Auditory implants. **(A)** Parts of the human ear. The outer ear comprises the pinna, the concha, and the ear canal. The middle ear components are the tympanic membrane and the three ossicular bones: malleus, incus, and stapes. The stapes connects the middle ear to the inner ear fluid-filled labyrinth through the oval window. The inner ear is divided into three regions: cochlea (which transduces sound through the auditory nerve to the auditory brain centers), vestibule, and semi-circular canals (chiefly associated with maintenance of balance). Please note that the components shown here are not drawn to scale. The purpose of this cartoon is solely to enhance the comprehension by the reader of the ear parts. **(B)** Uncoiled cochlea. The cochlea is divided into three compartments (scala vestibuli, scala media, and scala tympani) by two membranous structures: the vestibular membrane and the basilar membrane. The scala media contains endolymph and houses the Organ of Corti, which rests on the basilar membrane. The Organ of Corti contains sensory cells, which synapse with auditory nerve and are responsible for sound transmission to the auditory regions of the brain. Please note that here the figure parts are also not drawn to scale. **(C)** Cochlear implant (CI). The CI consists of an electrode that is introduced to the cochlea through the round window. This electrode is designed to maintain the tonotopism of an intact cochlea. It receives electrical signal from a subcutaneous implant (receiver) and serves to directly stimulate the auditory nerve. Please note that here that the figure parts are also not drawn to scale. **(D)** Auditory brain stem implant (ABI). This system consists of an electrode array implanted in the brain stem that collects signals from a receiver, which receives transmitted signals from an external processor. The external processor converts sound waves obtained from the integrated microphone into electrical energy and transmits it to the receiver. Figure parts are not drawn to scale.

The ear is analogous to a biological microphone. While a microphone converts sound vibrations to electric signals, the ear converts vibrations into nerve impulses that are processed by auditory regions in the brain (Alberti, 2021). The human ear is divided into three regions: outer ear, middle ear, and inner ear (Figure 2A). The outer ear consists of the pinna/auricle and ear funnel-like portion (the concha), and the external auditory canal (meatus); it functions by collecting sound and channelizing it to the tympanic membrane, a part of the middle ear. The middle ear is a moist air-filled cavity that extends to the pharynx through the Eustachian tube. The arrangement allows for pressure on either side of the eardrum to be equalized to atmospheric pressure. The middle ear houses three bones, the malleus, the incus and, the stapes, which connect the tympanic membrane to the oval window of the cochlea, which is a fluid-filled labyrinth. The middle ear acts as a transformer device, and it permits sound waves traveling in air to reach the inner ear without deflections. Without the middle ear, the efficiency of sound transmission would reduce by a factor of 30 (Roberts, 2002). The inner ear is divided into three regions: vestibule (expanded portion nearest the middle ear), semi-circular canals (three tubes situated in the three planes of space), and cochlea. Cochlear components transduce the sound vibrations received from the middle ear into nerve impulses that travel through the cochlear nerve to the auditory center in the brain. The cochlea is a coiled structure that is internally divided into three compartments (Figure 2B): scala vestibula, scala media, and scala tympani. Scala vestibula and scala tympani are connected at the helicotrema and contain a fluid called the perilymph (which has a composition similar to the cerebrospinal fluid and a high Na + content). The scala media is bound by the vestibular membrane and basilar membrane, and contains a fluid called endolymph (high K^+^ content) whose composition differs from that of the perilymph. The Organ of Corti is a specialized epithelium located on the basilar membrane in the scala media. It contains sensory and non-sensory cells. Sensory cells are termed as hair cells, since they contain hair-like protrusions (stereocilia) on their apical surface. The cochlea of humans contain 3,500 inner hair cells and 12,000 outer hair cells distributed in rows along the length of the cochlea (Roberts, 2002). Inner hairs are primary sensory cells, and they synapse with the cochlear (auditory) nerve. Another membrane called the tectorial membrane lies over the apical surface of the Organ of Corti and contacts the tips of the outer hair cell stereocilia (Roberts, 2002). As the two membranes, the tectorial and basilar membranes, move in response to the sound vibrations received from the middle ear at the oval window, the stereocilia also move, causing activation or deactivation of receptors on their hair cell surface. The cells depolarize causing calcium channels to open, which leads to calcium influx and subsequent release of a neurotransmitter, which then carries the impulse through the auditory nerve to auditory brain centers in the temporal lobe. The variation in rigidity and size of the hair cell that is arranged throughout the cochlea enables hair cells to respond to a range of frequencies from low to high. Cells at the cochlear apex respond to lower frequencies, while hair cells at the base of the cochlea (near the oval window) respond to higher frequencies, creating a tonotopic gradient throughout the cochlea (White et al., 2021).

The auditory system may fail in certain circumstances, leading to hearing loss. Hearing loss may be conductive, sensorineural, or mixed. In conductive hearing loss, vibrations from the outer ear do not reach the inner ear. Sensorineural hearing loss occurs when there is a defect in the inner ear. In mixed hearing loss, there is a combination of conductive and sensorineural components (Alshuaib et al., 2015). Hearing aids or implants may be employed to amend a dysfunctional auditory system. Hearing aids are sound-amplifying devices that enable the user to detect noise. Conventional hearing aids are non-invasive and are worn behind the pinna, in the ear canal, or in any other body location. The components of hearing aids are a microphone (converts sound energy into electrical energy), an amplifier (amplifies the input), a receiver (converts the amplified input back into acoustic energy and projects the waves to the ear canal), and a battery (to drive the device). Non-invasive hearing aids aim to increase sound levels to aid acoustic perception by inner hair cells in the cochlea (Schuster-Bruce and Gosnell, 2020). There are four types of hearing implants (which are surgically placed): middle ear implants (Haynes et al., 2009; Channer et al., 2011), cochlear implants, bone conduction hearing implants (Banga et al., 2011; Rohani et al., 2020), and auditory brainstem implants (ABI). Cochlear and ABI will be discussed here, since they are neuromodulatory in nature.

Cochlear implants can aid hearing in patients with sensorineural hearing loss. CIs bypass the eardrum, ossicular chain, basilar membrane, and dead hair cells. They stimulate auditory nerve fibers through electrodes implanted in the cochlea and are beneficial in patients with sensorineural hearing loss but with functional auditory nerve fibers (Svirsky, 2017). CIs may be totally implantable or may have external components (external microphone, processor, and transmitting coil) for empowering electrodes (Khosravi et al., 2018). In totally implantable CIs, the entire system is located underneath the skin. Sound signals in CIs, received by microphones (usually located behind the pinna), are processed and transmitted *via* radio frequency to a subcutaneous element that is surgically implanted in the temporal bone. The subcutaneous element then generates a train of pulses that are sent to an electrode array implanted in the cochlea (Figure 2C) through the round window, thus directly stimulating the auditory nerve fibers, and bypassing damaged hair cells of the cochlea (Calero et al., 2018). The electrode array inserted into the scala tympani of the cochlea may have 16–22 sites of stimulation, although there are no improvements in speech perception when the number of sites exceed 8 (Wilson, 2017). The speech processor filters the acoustic signal into several frequency bands that correspond to the normal range of speech sounds (Svirsky, 2017). The output of each frequency band is sent to different electrodes of the CI. High-frequency bands are intercepted by electrodes near the base of the cochlea, while low-frequency ones are caught by the electrodes in apical locations (Svirsky, 2017). The CI attempts to preserve the tonotopic map of the cochlea, and the brain perceives the received electrical impulses as sound (Vermeire et al., 2008). CIs may be implanted in a single ear (unilateral implantation) or in both ears (bilateral implantation). Compared with those patients who underwent unilateral cochlear implantation, patients with bilateral CIs have better auditory perception and can effectively localize the source of sound (Eapen et al., 2009; Xue et al., 2009; Khosravi et al., 2018). However, the cost of implants, along with complications associated with the implantation, outweighs the benefits of bilateral implantation (Xue et al., 2009). The surgical procedure for placement of CI is safe, although there are chances of post-implant complications, which may be categorized as minor or major complications (Venail et al., 2008; Farinetti et al., 2014; Khosravi et al., 2018). Major complications include death, meningitis, surgery without reimplantation in cases (scalp necrosis, severe infection, electrode shifting, eardrum perforation, receiver repositioning, and cholesteatoma), tinnitus, facial stimulation, and pain that cannot not be alleviated by electrode deactivation (Venail et al., 2008). Minor complications would be transient facial palsy, scalp hematoma, infections that resolve without recourse to surgery, and tinnitus, facial stimulation, and pain that can be relieved by electrode deactivation (Venail et al., 2008). CIs are indicated for patients with cochlear sensorineural losses. These are identified by their unresponsiveness to sound stimulation by the most powerful hearing aids. Additionally, children below the age of 6 suffering from congenital or acquired profound hearing loss but with an intact auditory nerve benefit from CIs. Adults who have learned languages prior to the acoustic loss are also ideal candidates (Kameswaram and Raghunandhan, 2013). Congenitally deaf children would benefit from the implantation of CIs before the age of 3, since this would help them gain language proficiency that would be instrumental in their speech development (Svirsky, 2017). Adults with acquired hearing loss need to be counseled before implantation, so that they are not disillusioned when the implant derived auditory perception differs from normal acoustic hearing. As of 2019, over half a million people from the age group of 6–90 years have CIs. CIs have enabled users to use the telephone, understand speech in the presence of background noise, and has released them from the isolation of deafness (Boyle, 2020). Although CIs cannot replace the normal auditory system, CI implant wearers can communicate effectively with less effort compared with their counterparts without a device (Boyle, 2020). A recent CI is the Nucleus 24 Cochlear Implant System by Cochlear Americas; it gained United States Food and Drug Administration (FDA) approval in March 2020.

In patients with a missing or small auditory nerve, or severely abnormal cochlea, an ABI would be beneficial. These implants can stimulate hearing pathways in the brain stem bypassing the inner ear and auditory nerve. Candidates for ABI are patients diagnosed with neurofibromatosis 2 (NF2), children with cochlear nerve aplasia, and post-meningitis patients with ossified cochlea (Bento et al., 2008). In patients with NF2, bilateral acoustic neuromas (vestibular schwannomas) are observed, which compress the brain stem (Asthagiri et al., 2009; Colletti et al., 2012). When these are surgically removed, they typically cause disruption to the cochlear nerves. In 2000, ABIs were approved by the United States FDA for clinical use in patients with NF2 (Colletti et al., 2012). A clinical study on the feasibility of an ABI for children with no cochlea or auditory nerve (NCT02310399) and without NF2 is also being conducted. However, two clinical trials on the suitability of ABIs in adults (NCT01736267) and pediatric (NCT01864291) subjects with NF2 were discontinued. An ABI (Figure 2D) consists of an external device and an internal receiver-stimulator implant. The external device consists of a battery source, microphone, speech processor, transmitter coil, and a magnet worn above and behind the ear. The internal component consists of a receiver-stimulator and magnet, a ground electrode, and an electrode array, which are all embedded in a flat and rigid conformation along the highly curved surface of the cochlear nucleus in the brain stem (Wong et al., 2019). The Nucleus 24 ABI system or ABI24 consists of 21.7-mm platinum disk electrodes (for stimulation of the brain stem) aligned on a silicone and mesh backing (Schwartz et al., 2008). Initial stimulation employing an ABI is performed 6–8 weeks following implantation. The programming of ABIs is far more complex than that of CIs because of variable pitch precepts and generation of non-auditory responses (caused by the spread of stimulation beyond the cochlear nuclear complex). Thus, initial stimulation involves setting of thresholds, evaluation of patient comfort level, management of non-auditory stimulation, and pitch scaling (Schwartz et al., 2008). Modern CIs offer much better speech perception than ABIs; however, auditory sensations provided by ABIs are beneficial when used in combination with lip-reading, and facilitate oral communication (Schwartz et al., 2008). Most patients with NF2 benefited from ABIs, since they received auditory inputs that are essential in everyday life. They could detect and discriminate distinct sounds, e.g., dog barking, phone ringing. They were more effective at lip reading, and there was considerable improvement in communication. However, ABI users could identify few words using the auditory sensations provided by the device alone (not more that 20% of words in simple sentences; Colletti et al., 2012).

Batuk et al. (2020) investigated the bimodal stimulation of children who have inner ear malformation and found that the coherent use of CIs and ABIs increased performance and speech perception. In another study on the efficacy of ABIs, for improvement of speech perception and audiometry tests in patients with post meningitis hearing loss, there was an increase in hearing performance post implantation of the device (Malerbi et al., 2018).

### Neural Stimulators

Neural simulation can be divided into central nervous system (CNS) stimulation (deep brain simulation and spinal cord simulation) and peripheral nervous system (PNS) stimulation (vagus nerve stimulation and sacral nerve stimulation).

#### Deep Brain Stimulation

Deep brain stimulation (DBS) involves the application of high-frequency (above 100 Hz, typically 130–185 Hz; Ramasubbu et al., 2018) electrical stimulation to deep brain structures. The idea of DBS for the treatment of movement disorders originated from the study of Benabid et al. (1991, 1994, 1996), which showed that it was possible to alter the function of neuronal tissue with electricity. DBS, targeting internal structures of the globus pallidus internus, subthalamic nucleus, or ventral intermedius nucleus of the thalamus (Vim), is performed to treat Parkinson’s disease (PD; Fitzgerald, 2018) and several forms of tremor and dystonia (Chiken and Nambu, 2016; Ashkan et al., 2017; Jakobs et al., 2019). DBS is a neuromodulatory technique that modifies neural function through the application of electrical current (Jakobs et al., 2019). The most used DBS system has a four-contact stimulating electrode that is stereotactically implanted in deep brain structures and connected *via* a subcutaneous wire to a pacemaker-like unit called implantable pulse generator (IPG) that is placed on the chest wall underneath the collarbone (Herrington et al., 2016). A clinician uses a portable external device that communicates wirelessly with the IPG to adjust the parameters of stimulation to maximize symptomatic relief and minimize side effects (Herrington et al., 2016). The mechanism of DBS depends on the site of stimulation and applied frequency. The effect of stimulation on a particular tissue depends on the fraction of cell bodies and axons, types of ion channels on the cell soma and axon, diameter of axons, degree of myelination of axons, orientation of axons with respect to the electrode, distance from the electrode, and microenvironment (presence of microglia, astrocytes) (Jakobs et al., 2019). Furthermore, the effects of stimulation are dependent on device parameters, such as single pulse or continuous stimulation, amplitude, voltage, polarity, frequency, pulse width, pulse shape, and rhythm (Jakobs et al., 2019). Greatest relief of symptoms was obtained at stimulation frequencies >100 Hz, and no therapeutic relief was found at frequencies <50 Hz. Longer pulses of stimulation exerted their influence on cell bodies, while shorter pulses affected axons. DBS is localized, since small currents are generated (2 mA spreading over 2–3 mm), and the intensity of stimulation decreases proportionally to the square of the distance from the source (Kern and Kumar, 2007). Some proposed theories regarding the mechanism of DBS are high frequency stimulation-induced blockage of voltage-gated current (Beurrier et al., 2001), inhibition of intrinsic neuronal firing followed by its replacement with a high-frequency regular pattern output (Filali et al., 2004), and depression of synaptic transmission due to neurotransmitter depletion (Gang et al., 2005). DBS and ablation therapy are indicated for tremor and PD. DBS may be a preferred treatment compared with lesioning, as less brain tissue is damaged. Furthermore, in DBS, parameters may be adjusted for optimal effects, and the electrode may surgically repositioned or removed if required. Lesioning, on the other hand, is a permanent (Kern and Kumar, 2007) alteration. DBS complications are related to the hardware, stimulation-induced side effects, and interference with stimulation from external electromagnetic fields (Blomstedt and Hariz, 2006). Postoperatively, problems with DBS would be breakage of the hardware, skin infections, and skin erosion over the implanted hardware (Kern and Kumar, 2007). DBS is expensive compared with lesioning, and the IPG must be replaced at intervals of approximately 4–7 years (Blomstedt and Hariz, 2006). DBS is currently FDA-approved for the treatment of PD, essential tremor (ET), dystonia, obsessive-compulsive disorder, and medically refractory epilepsy (Lee et al., 2019). As of 2019, 16,000 patients worldwide have received DBS, and the number continues to increase by 12,000/year (Lee et al., 2019). Other disorders currently under clinical investigation with DBS are major depression, tinnitus, Tourette syndrome, Alzheimer’s disease (AD), pain (phantom pain, deafferentation pain, central pain, and nociceptive pain), addiction, and anorexia nervosa (Lozano et al., 2019).

#### Vagus Nerve Stimulation

The vagus nerve (cranial nerve X) is a mixed nerve composed of 20% efferent (motor) nerve fibers and 80% of afferent (sensory) nerve fibers. Efferent nerve fibers are cholinergic and form a part of the parasympathetic autonomous nervous system. Branches of vagus nerve innervate the larynx, pharynx, heart, lungs, and organs associated with the gastrointestinal tract (Howland, 2014). The light and left vagus nerves exit from the brain stem, course along the length of the neck, upper chest, lower chest, diaphragm, and into the abdominal cavity. Vagus nerve stimulation (VNS) refers to as any method that causes stimulation of the nerve. The cervical vagus is a popular clinical target for invasive neuromodulation due to easy surgical implantation of device components. At the cervical level, the vagus nerve contains several types of fibers with respect to size, myelination, and direction (afferent and efferent; Ahmed et al., 2020). Cervical VNS is being investigated as treatment for several disorders, such as drug-resistant epilepsy, depression, AD, anxiety, pain, tinnitus, sepsis, rheumatoid arthritis, heart failure, diabetes, and obesity (Johnson and Wilson, 2018; Chang et al., 2020). It is suggested that the anti-epileptic action is mediated through afferent large myelinated-A fibers, cardiac function modulation through efferent myelinated B fibers, anti-inflammatory action via B fibers, and *via* afferent unmyelinated C-fibers (Chang et al., 2020). VNS of vagal afferents at a frequency of 20–30 Hz has been performed to treat refractory epilepsy and depression (Bonaz et al., 2013). Low-frequency (5 Hz) stimulation of vagus efferents is found to activate the cholinergic anti-inflammatory pathway (CAP; Bonaz et al., 2013) and is also being investigated in cardiac therapy (Wang et al., 2019). Stimulation of vagus efferent nerve fibers has been associated with slowing of heart rate, induction of gastric motility, dilation of arterioles, constriction of pupils, and inhibition of the inflammatory response (Tracey, 2002; Breit et al., 2018).

Electrical stimulation of the left cervical vagus nerve through an implanted device was approved by the United States FDA for the treatment of refractory epilepsy in 1997, and was approved for treatment of chronic treatment resistant depression in 2005 (Howland, 2014). The VNS therapy for the above-mentioned indications has since been approved in more than 70 countries, and more than 1,00,000 patients have received the therapy (Wheless et al., 2018). VNS consists of a pulse generator placed on the left upper chest that is attached to a lead wire that is subcutaneously implanted and connects the bipolar lead attached to the left mid-cervical vagus nerve (surgically placed through an incision in the left neck area; Howland, 2014; Wheless et al., 2018). The system also comprises a handheld computer, a tunneling tool, and handheld magnets. The handheld computer programs the pulse generator (which sends electrical signals to the vagus nerve through the lead) *via* a programming wand placed on the skin over the device. Programmable parameters that are adjusted in therapy are the current intensity (0.5–3.5 mA), the frequency (20–30 Hz), pulse width (500 μs), and the stimulation on time (30–90 s) followed by an off time of 5 min (Groves and Brown, 2005; Bonaz et al., 2013). The VNS system can be turned off and on by the programmer and can be shut temporarily by holding a magnet over the device (Howland, 2014). Models of VNS therapy generators (from LivaNova, USA, Inc., Houston, TX, United States) are Demipulse Model 103, Demipulse DuoMode 104, AspireHC Model 105, AspireSR Model 106, and Sentiva Model 1000. The available leads (from LivaNova, USA, Inc., Houston, TX, United States) are Perennia Model 303 and Perennia FLEX Model 304 (Wheless et al., 2018).

Abnormalities in the functioning of the autonomic nervous system, such as sustained sympathetic overdrive and parasympathetic withdrawal, are some of the characteristic features of heart failure (HF). In HF, due to sympathovagal imbalance, the heart rate increases, there is excessive release of pro-inflammatory cytokines, dysregulation of nitric oxide pathways, and arrythmias (Sabbah, 2018). Studies reported that alteration in cardiac vagal efferent activity could decrease heart rate and improve ventricular contractile function, thus paving the way for electrical cervical VNS therapy to prevent sudden cardiac death and improve long-term survival (Sabbah, 2018). Right cervical vagus nerve-based implants for VNS have been tested clinically for the treatment of congestive heart failure. In a previous clinical trial, INcrease Of VAgal TonE in Heart Failure (INOVATE-HF)-NCT01303718, a BioControl CardioFit system was used. In this system, a standard transcutaneous lead was placed in the right ventricle of patients for sensing ventricular activation, and a nerve stimulator cuff was placed on the right vagus nerve. The trial, however, did not produce conclusive evidence that VNS could reduce the risk of death or HF events among patients with HF and reduced left ventricular ejection fraction (Gold et al., 2016). Other clinical trials that were conducted were NEuroCardiac TherApy fOR Heart Failure (NECTAR –HF-NCT01385176) and The Autonomic Regulation Therapy for the Improvement of Left Ventricular Function and Heart Failure Symptoms (ANTHEM-HF). NECTAR-HF used a device to stimulate the right cervical vagus nerve and intended to compare VNS with medical therapy in symptomatic patients with HF and those with severe left ventricular systolic function. The delivered VNS improved the quality of life of patients but failed to show an improvement in left ventricular systolic-end diameter, which was the primary endpoint of the trail (Zannad et al., 2015). The ANTHEM-HF study enrolled 60 subjects with class II–III HF with left ventricular ejection fraction < 40%. The patients received VNS devices (Demipulse Model 103 pulse generator and PerenniaFLEX Model 304 lead, Cyberonics, Houstan TX, United States) with lead placement to either the right or left cervical vagus nerve. The trial concluded that right-sided or left-sided VNS was feasible and well-tolerated in patients with improvements in cardiac function and HF symptoms (Premchand et al., 2014, 2016). The parameters used in all these three trials were different (Anand et al., 2019). The ANTHEM-HF trial led to the larger Autonomic Regulation Therapy to Enhance Myocardial Function and Reduce Progression of Heart Failure with Reduced Ejection Fraction (ANTHEM-HFrEF-NCT03425422) trial. This study is being conducted by LivaNova and uses the device VITARIA, which is implanted on the right cervical vagus nerve for stimulation purposes.

Invasive VNS, in which an implant is surgically placed on the left or right cervical vagus nerve, is an expensive treatment and may involve the occurrence of adverse events during the procedure. Complications that may arise during and directly after implantation are common infections, vocal cord pareses, lower facial weakness, bradycardia, and asystole (Ben-Menachem, 2001). Non-invasive or transcutaneous VNS strategies are being studied as alterative techniques against certain conditions, such as depression, epilepsy, tinnitus (Stegeman et al., 2021), migraine (Silberstein et al., 2020), and pain (Yap et al., 2020). Transcutaneous VNS can be further categorized as transauricular VNS (taVNS) or transcervical VNS (tcVNS). The basis of taVNS is that the outer ear (tragus, concha, and cymba concha) is innervated by afferent fibers of the auditory branch of the vagus nerve (ABVN; Peuker and Filler, 2002). There are speculations, however, that the tragus is not majorly innervated by ABVN; therefore, it may not be the ideal location for taVNS (Badran et al., 2018; Burger and Verkuil, 2018) compared to the cymba concha. Stimulation of the ABVN *via* the cymba concha was found to activate the nucleus tractii solitarii (NTS), which relays information from vagal afferents to higher-order vagal projections in the brainstem and forebrain (Frangos et al., 2015). However, a recent review suggests that the NTS can also be activated *via* non-vagal routes (Cakmak, 2019). The NTS is suggested to be engaged in the control of respiration, mediation of blood pressure and heart rate, mediation of emotional response, and contribution to memory (AbuAlrob and Tadi, 2020).

Electrical stimulation *via* taVNS might be advantageous for treatment of major depressive disorder and inflammation associated with depressive episodes (Liu et al., 2020). A taVNS device, NEMOS, applied to the concha (Cerbomed GmbH, Erlangen, Germany), received European clearance for treatment of epilepsy, depression, and pain (Howland, 2014). Low-level taVNS to the tragus region of the ear was studied in a randomized clinical trial (NCT02548754) named Transcutaneous Electrical Vagus Nerve Stimulation to Suppress Atrial Fibrillation (TREAT-AF). The study showed that chronic, intermittent taVNS of the ABVN at the tragus suppressed atrial fibrillation (AF) in patients with paroxysmal AF over a 6-month period (Stavrakis et al., 2020). In another study, taVNS to ABVN through the auricular concha was used in a clinical trial in China (ChiCTR-TRC-13003519) for the treatment of insomnia. taVNS was found to relieve insomnia over a 4-week treatment period and could ameliorate fatigue and improve the quality of life of participants by reducing the concomitant symptoms of depression and anxiety (Jiao et al., 2020). The Neuromodulation to Regulate Inflammation and Autonomic Imbalance in Sepsis (NERINA-SEPSIS) clinical trial (NCT03992378) is investigating low-level taVNS through the tragus for treatment of sepsis. The central hypothesis of this pilot clinical trial is that taVNS at the tragus of the external ear can activate the CAP to suppress inflammation and improve autonomic imbalance as measured by inflammatory cytokine levels and heart rate variability analysis. Readers are prompted to refer to the review by Kaniusas et al. (2019), which provides a physiological perspective of taVNS and its implications for treating several disorders.

A tcVNS device called gammaCore^®^ (Electrocore, Basking Ridge, NJ, United States) has been successful in treating patients with episodes of migraine (Kinfe et al., 2015) and has been approved by the FDA for primary headache (Mwamburi et al., 2020). The device is designed to stimulate myelinated sensory afferent left cervical vagus nerves through the neck. For tcVNS, left cervical VNS is preferred over right cervical VNS, since right-sided VNS may result in bradycardia (Yuan and Silberstein, 2016; Yap et al., 2020), which may complicate therapy. gammaCore-based short term tcVNS was investigated in a small cohort of patients with refractory gastroparesis (a chronic motility disorder that delays gastric emptying time in the absence of mechanical obstruction), with some patients responding positively to the treatment (Paulon et al., 2017). Implantation of a gastric electric stimulation device is usually the treatment offered to patients with gastroparesis who do not respond to pharmacological treatment, so tcVNS is offered as a suitable alternative. The parent company of gammaCore^®^, ElectroCore, is involved in active research to find other conditions in which the device can be used as a therapeutic intervention.

Side effects seen in long-term treatment with VNS implantation are cough, voice alteration, dyspnea, pain, paresthesia, headache, pharyngitis, depression, infection, and death (Ben-Menachem, 2001, 2002). The vagus nerve projects from the brain to several organs in the thorax and abdomen, such as the heart, lungs, larynx, pharynx, stomach, spleen, pancreas, liver, intestines, and ovaries. Moreover, the vagus nerve contains bundles of fibers that vary in diameter and conduction velocity. These fibers are activated in order of their size from the largest (A fibers) to the smallest (C fibers). Since VNS is not selective, it is associated with the side effects mentioned above because of the activation of off-target nerve bundles. Selective VNS (sVNS) is an emerging field where attempts are being made to selectively activate the nerve fibers of interest. Evolving strategies in the design of vagus nerve stimulators to achieve selectivity are fiber-selective stimulation, spatially selective stimulation, anodal block, kilohertz electrical stimulation block, and neural titration (Fitchett et al., 2021). An overview of these designs for sVNS is well illustrated by Fitchett et al. (2021), and is recommended to readers.

#### Spinal Cord Stimulation

Spinal cord stimulation was proposed in 1967 by Shealy et al. (1967) as an alternative to neuroablation in pain therapy. Main indications for spinal cord stimulation are vascular pain, rachidian pain, chronic regional pain syndrome, neuropathic perineal pain, and pain due to urological diseases (Constantini, 2005). In a spinal cord stimulation (SCS) system, there are two basic components: an epidural electrode (lead) and an IPG (Hegarty, 2012). Spinal cord stimulator electrodes are placed in the cervical (C3-4, C5-7), thoracic (T3 through T12), thoracolumbar, and lumbar regions (T11-L1, T12-L1, L1-L2) of the spinal cord (Zan et al., 2011) based on the type of pain treated. Depending on the frequency of the stimulating electrical energy and duration of the pulse, SCS can be differentiated into tonic SCS, high frequency SCS, burst SCS, or closed loop SCS (Heijmans and Joosten, 2020). Although the targets of all these SCS methods are under investigation, the concept of pain suppression remains similar. It involves the activation of large-diameter afferent fibers leading to the release of neurotransmitters that close the gate that permits the transmission of pain signals to the brain.

Spinal cord stimulation has also been investigated for treatment of HF in humans. Two clinical trials, The SCS for Heart Failure (SCS HEART) and Determining the Feasibility of Spinal Cord Neuromodulation for Treatment of Chronic Heart Failure (DEFEAT-HF) were conducted. Although the results of the trials were promising, the sample size of the trails was too small to confirm the efficacy of SCS for HF (Tse and Schwartz, 2017).

Besides pain management and HF, other emerging conditions that are candidates for SCS therapy are PD, spasticity, and spinal trauma rehabilitation (Tapias Pérez, 2021). Transcutaneous SCS is an evolving non-invasive method of neuronal stimulation explored for the treatment of spasticity after spinal cord injury (Hofstoetter et al., 2020). A clinical trial is currently being conducted to compare epidural SCS versus transcutaneous SCS on patients with incomplete and chronic spinal cord injuries (NCT04043715). The trial measures the improvement in locomotion of patients following the stimulation of lumbosacral circuits by SCS. Transcutaneous SCS is also being investigated for treatment of freezing of gait in patients with PD (Reis Menezes et al., 2020).

#### Sacral Nerve Stimulation

Sacral nerve stimulation (SNS) is the stimulation of the sacral nerve to modulate neural reflexes that influence the bladder, sphincter, and pelvic region (Wein and Moy, 2007). The implantable system for sacral nerve neuromodulation consists of a neurostimulator, a wired cable, and a lead with quadripole electrodes. The electrode is inserted in one of the sacral foramina (typically at S3), and the neurostimulator is implanted subcutaneously in the lower quadrant of the abdomen. A physician can modify the parameters of stimulation through a remote electronic programmer. A magnet is provided to patients to turn the neurostimulator on or off and adjust the level of stimulation (Chancellor and Chartier-Kastler, 2000; Blok, 2018). The United States FDA has approved sacral neural modulation for three indications: urge incontinence, urge frequency, and non-obstructive urinary retention. The indications have recently expanded to include other voiding disorders such as interstitial cystitis/painful bladder syndrome (Elkelini et al., 2010). SNS systems, such as InterStim^TM^ (Medtronics Inc.) are available in the market.

Studies are underway to test sacral nerve modulation strategies for patients with functional bowel disorders, such as gastroparesis, functional dyspepsia, gastroesophageal reflux, fecal incontinence, and constipation, that are not responsive to maximal medical treatment, bowel lavage, or biofeedback therapy (Raina, 2020).

### *In vitro* Bioelectronic Devices Based on Neurosensory Principles

#### Bioelectronic Nose

The sense of smell or olfaction is the result of the development of action potentials that arise in olfactory sensory neurons (OSNs) of the nasal passage olfactory epithelium and are transmitted *via* the olfactory nerve to the olfactory bulb and then to the olfactory cortex in the brain (Sharma and Matsunami, 2014; Branigan and Tadi, 2021). OSNs are bipolar neurons whose axons bundle up to form the olfactory nerve while their dendrites form knob-like structures at the junction between the olfactory epithelia and nasal passage. These knob-like dendritic structures have cilia or olfactory hair immersed in the nasal mucus, in which odorants dissolve. When dissolved odorants encounter olfactory receptors (which belong to the family of G-PCRs) on the cilia, they cause depolarization of cells, which is propagated along axons. Humans have the propensity of identifying several odors resulting from the activation of different combinations of olfactory receptors (Sharma and Matsunami, 2014; Branigan and Tadi, 2021). Humans can recognize odorant molecules at the concentration of 10^–3^ ppb and detect around 10,000 different odor molecules even though the number of functional olfactory receptors is about 390 (Ko et al., 2018).

Biomimetic systems that employ biological components to mimic the detection of odors have been constructed and termed as bioelectronic noses (BENs). A BEN consists of two main parts: a primary transducer and a secondary transducer. Biological components comprise the primary transducer, while the secondary transducer is the non-biological component, which is responsible for device sensitivity. Biological materials that are used to construct the primary transducer are cells, proteins, nanovesicles, and peptides. Secondary transducers could be based on surface plasmon resonance, quartz crystal microbalance, carbon nanotube field effect transistor, carboxylated PPy, nanotube field effect transistor, and graphene-based field effect transistor devices (Ko and Park, 2016; Ko et al., 2018). Enzymes, aptamers, and antibodies can also be used as biological components, but they may not be efficient in detecting all the different odors that a living mammal can easily decipher. Scientists have tried to emulate the mammalian olfactory mechanisms by including the olfactory tissue, the olfactory receptor cells, or the olfactory receptors themselves as primary transducers. In an example, cell-based BENs were constructed using a light-addressable potentiometric sensor (LAPS) as the secondary transducer (Liu et al., 2006). Briefly, the LAPS silicon surface was coated with laminin and poly L-ornithine to promote attachment of cells (the olfactory receptor neurons and olfactory bulb cells obtained from 5- to 7-day-old rat pups), which were seeded on a chip. At the end of 7 days of seeding, neural networks were formed. The BEN was successfully tested to detect acetic acid at concentrations of 1, 25, and 50 μM (Liu et al., 2006). However, cell-based systems are difficult to maintain in a viable state, are too large for a nanomaterial-based sensor platform, and contribute significantly to noise (non-odor-related signals) due to cellular metabolism (Ko et al., 2018). Non-cellular alternatives are being investigated, such as olfactory receptors and nanovesicles derived from engineered olfactory cells which can emulate the olfactory system (Ko and Park, 2016). Self-assembled monolayer (SAM) is one of the techniques that have been utilized to immobilize engineered olfactory receptors to sensors (Wu et al., 2012). SAMs supposedly significantly improve immobilization efficiencies by providing an ultra-thin functional layer for biomolecule immobilization. In an example, bioengineered olfactory receptor ODR-10 from *Caenorhabditis elegans* was expressed in human breast cancer MCF-7 cells and isolated as membrane fractions. These were immobilized on 16-Mercaptohexadecanoic acid [MHDA]-based SAMs that were activated with 1-ethyl-3-(3 dimethylaminopropyl (EDC) carbodiimide and N-hydroxy succinimide to enable covalent coupling of the ODR-10 membrane fractions to the SAMs. The SAMs in turn, coated a surface acoustic wave sensor, which worked as a secondary transducer. The device could successfully detect diacetyl (a specific odorant of ODR-10, a butter flavor substance) in the range of 10^–4^ to 10^–10^ M (Wu et al., 2012). In another study, the ODR-10 from *C. elegans* was overexpressed in *Escherichia coli* purified, stabilized in a micellar structure, and then immobilized on a secondary transducer, a carbon nanotube field effect transistor (Shin et al., 2020). The BEN could detect diacetyl levels (detection limit 10^–15^ M) in beer and wine, and served in quality control of these beverages. In another example, olfactory receptor-derived peptides were used as a primary transducer for the construction of a peptide receptor based bioelectronic nose, using single-walled carbon nanotube field effect transistors as the secondary transducer (Lim et al., 2013). This device could detect trimethylamine that emanates from spoiled sea food at a detection limit of 10^–15^ M and could discriminate it from other molecules in real time (Lim et al., 2013). A BEN using nanovesicles of human olfactory receptors (hORs; overexpressed in HEK-293 cells) was constructed using single-walled nanotube-based field effect transistors as the sensory element. The BEN was constructed with the hORs that were specific for detection of heptanal (a biomarker for small cell lung cancer) in blood. The device could detect heptanal at a concentration of 10^–14^ M in real time (Lim et al., 2014). BENs find application in various fields as diagnostic devices in medicine, devices employed in food quality control, in environmental monitoring, and for smell visualization (Dung et al., 2018).

#### Bioelectronic Tongue

The widely accepted definition of electronic tongue systems states, “The electronic tongue is an analytical instrument comprising an array of non-specific, low-selective, chemical sensors with high stability and cross-sensitivity to different species in solution and an appropriate method of pattern recognition and/or multivariate calibration for data processing” (Vlasov et al., 2005). Few electronic tongue systems have been commercialized, such as SA402B and TS-500Z Taste Sensing System (Intelligent Sensor Technology, Inc., Atsug-shi, Kangawa, Japan) consisting of seven potentiometric electrode and lipid polymeric membranes, Astree II (Alpha, MOS, Toule France), composed of seven-ion selective field effect transistors, the Multiarray Chemical Sensor (McScience Inc. Suwon, South Korea) built with PVC and polyurethane membranes, and Sensor System (St. Petersburg, Russia) composed of seven potentiometric ion-selective sensors (Podrazka et al., 2017).

Bioelectronic tongues (BETs) are a variant of ETs that employ single or an array of biosensors that can analyze chemical species when coupled to a chemometric tool for interpretation of acquired data. Thus BETs, in contrast to ETs, are highly specific because of the incorporation of a biological element for recognition. They contain a bio-sensing/bio-recognition element coupled to secondary transducers. While the biosensors simulate biological mechanisms of detection and recognition of selected substances, secondary transducers convert biological signals to analytical ones which are further processed by the device. Transducers are further classified based on voltametric/amperometric, potentiometric, and piezoelectric principles. The modeling of the device (BET) to measure the multivariate response utilizes artificial neural network (ANN)-based chemometric procedures, such as pattern recognition, principal component analysis, and multivariate analysis (Wasilewski et al., 2020). Based on the biorecognition element, BETs can be categorized as tissue-based, cell-based, taste receptor-based, enzymatic, antibody-based, molecularly imprinted polymers, or peptide-based (Wasilewski et al., 2020). Enzymes are most commonly used as the biorecognition element in BETs as enzymatic single-channel biosensors, and some examples of enzymes employed are tyrosinase from mushrooms and laccase from *Agaricus bisporus* for detection of phenols, glucose oxidase from *Aspergillus niger* for glucose detection, and fructose dehydrogenase for detection of D-fructose (Skládal, 2020). Enzyme-based BETs may suffer from inhibition of enzyme activity due to presence of other externally added substrates, and presence of inhibitors (Skládal, 2020). Antibody-based BETs may be used if the analyte is known and requires complex labeling strategies and exhibits high specificity for the analyte alone. A US patent (US2010/0222224) obtained for a BET for food allergy detection employs antibodies as the biorecognition element (Suni et al., 2010). Molecular imprinted polymers may be used as an alternative to antibodies because of their stability, robustness, low production cost, easily tunable selectivity, and comparatively weaker affinity for analytes, which allows testing of many similar substances. Nucleic acid arrays, as biosensing materials for BETs, are not so popular, since they are expensive and require sophisticated bioinformatic tools to interpret the output obtained from the device (Skládal, 2020).

Taste (gustatory) receptors have also been investigated in the preparation of BETs. Physiological tastes are categorized as salty, acidic (sour), sweet, bitter, or umami (meaty taste attributed to amino acids such as glutamate in food). Some research investigates “fatty” and “metallic” taste as well. The different tastes are perceived by different ion channels, receptor molecules on the human tongue. Taste receptor cells are classified as Type I, Type II, or Type III cells. Type II cells contain G-protein-coupled receptors (GPCRs), which bind to sweet, bitter, and umami ligands, while Types III and I cells contain ion channels used in the detection of acidic (sour) and salty tastes, respectively (Chaudhari and Roper, 2010). The presence of tastants activates gustatory receptor cells, which get depolarized and, through a chain of intracellular events, cause the release of neurotransmitters at their synaptic junction with first-order neurons that form the initial part of the gustatory pathway. Through the nerve fibers of this pathway, the information is transmitted, and the taste is perceived by the brain.

Bioelectronic tongues composed of receptor proteins were prepared by Kim and colleagues (Kim et al., 2011; Song et al., 2014; Ahn et al., 2016). In an earlier study (Kim et al., 2011), they expressed a bitter human taste receptor protein in *E. coli* and immobilized the protein with its associated lipid membrane on single-welled carbon nanotube field-effect transistors to enable electrical monitoring of the receptor activity. The device could detect bitter tastants at 100 fM concentrations and could distinguish between bitter and non-bitter tastants of similar chemical structures. Then, the scientists developed a nanovesicle BET, which could detect sweeteners (Song et al., 2014). The human receptor for detection of sweet taste is a G-PCR, which is heterodimeric and composed of two receptor proteins, hTAS1R3 and hTASIR2. HEK-293 cells were engineered to express the human heterodimeric taste receptor composed of hTAS1R3 and hTAS1R2. The modified cells were grown and subsequently treated to enable the isolation of nanovesicles containing both the proteins and cell-signaling machineries, such as G protein, adenylyl cyclase, and ion channels. The nanovesicles were immobilized on single-welled carbon nanotube field-effect transistors to create a BET. The BET recognized various sweeteners, such as natural and artificial sweeteners, with high sensitivity and human-like broad specificity (Song et al., 2014). The same research group developed a duplex BET using graphene field-effect transistors conjugated to human taste receptor nanovesicles (Ahn et al., 2016). The human taste receptor nanovesicles were prepared by treating HEK-293 cells expressing three heterodimeric human taste receptors specific for sweet and umami tastants. The said device could successfully detect the two different categories of tastants and had the ability to detect the taste-enhancing effect as in the human sensory systems (Ahn et al., 2016).

Cell-based biosensing elements have also been investigated for the construction of BETs. Zhang and colleagues (Zhang et al., 2017) dissociated single receptor cells from primary taste cells obtained from fungiform papillae in the front tongue of female adult Sprague Dawley rats and immobilized them on a micro-electrode array (MEA) chip that was precoated with poly L-ornithine and laminin for better coupling. This BET was successfully explored for the detection of sour tastants (Zhang et al., 2017). The presence of taste receptors in tissues besides the tongue, such as those of the gastrointestinal tract, respiratory system, male reproductive system, brain, and heart have encouraged scientists to use these cell types for the construction of BETs. Rat cardiomyocytes were found to express bitter (Tas2r) and umami (Tas1r1, Tas1r3) taste receptors (Foster et al., 2013; Wei et al., 2019). Using this information, a bionic *in vitro* cell-based BET for the detection of bitter and umami tastants was constructed using rat cardiomyocytes as the taste sensing element and MEAs as the transducing element (Wei et al., 2019). The cardiomyocytes expressed only seven kinds of bitter tastant receptors; thus, many bitter compounds would not be detected by the device. However, the device could detect two bitter substances (Dena and Diph) and an umami compound (Mono sodium glutamate) at a concentration of 10^–6^ M (Wei et al., 2019).

Tissues of living organisms can be used as effective taste sensing components in a BET. As compared with cells, intact taste bud tissues can be easily obtained with preserved receptor structures (Wasilewski et al., 2020). In an example, tongues of Sprague Dawley rats were removed after the rats were sacrificed by decapitation. Taste-bud tissues were placed between two nuclear microporous membranes and fixed in sodium alginate-starch. The assembly was dipped into 5% calcium chloride for 10 s to form a sensing membrane, which was fixed on a pre-treated glass carbon electrode. Capsaicin and analgesic compounds were detected using this device; moreover, the device could also be used to screen analgesic compounds for their toxicity (Xiao et al., 2019).

Similar to electronic tongue applications, BETs can find wide applications in several food, brewery, and pharmaceutical industries for generating an authenticated organoleptic profile of the final product, from several production batches for consistency, safety, and predictability, and to ensure reproducibility (Di Rosa et al., 2020).

## Current Status and Future Scope

The future of BEMs based on neuromodulation is bright, and the scope is expanding as advances in device technology accelerate, and the unraveling of neural mechanisms in the treatment of several diseases are increasingly explored. Several devices are on clinical trials (Table 1), and there are many more at the preclinical, laboratory, or theoretical stage. The market for BEMs is large and is expected to step up in the coming decade. According to IDTechEx, BEM is at a 2.6 billion dollar market today and is predicted to exceed 60 billion dollars in 2029 (Alliance for Advancing Bioelectronic Medicine, 2020). It is estimated that the market for retinal implants and PNS [especially VNS (Pavlov and Tracey, 2019)] will far exceed the demand for CNS stimulation and cochlear implants (Alliance for Advancing Bioelectronic Medicine, 2020).

**TABLE 1:** Neuromodulating devices on clinical trials.

**S. no.**	**Implant**	**Sponsor**	**Status**	**Condition/Disease**	**References**
**Ear implants**					
1	CI532 cochlear implant and CP1000 (Nucleus 7)	Cochlear	Clinical Trial No. NCT03007472	Sensorineural Hearing Loss,	Kelsall et al., 2021
2	HiRes 90K^TM^ Advantage implant with HiFocus^TM^ Mid-Scala electrode. Electro-acoustic stimulation technology (EAS) sound processor	Advanced Bionics	Clinical Trial No. NCT02189798	Hearing Loss, Partial Deafness, Hearing Disorders, Ear Diseases, Otorhinolaryngologic Diseases	
**Neural stimulators**					
1	Non -Invasive Vagus nerve stimulant: gammaCore-S	ElectroCore INC	Clinical Trail No. NCT02686034	For acute treatment of migraine attacks	
2	Auricle Vagus Nerve Stimulator	Northwell Health	Clinical Trial No. NCT01569789 Phase I NCT00859859 Phase I	Inflammatory diseases such as rheumatoid arthritis	Addorisio et al., 2019
3	Transcutaneous Electrical Auricular Stimulator	Northwell Health	Clinical Trial No. NCT02910973	Prevention of release of inflammatory cytokines by harnessing cholinergic anti-inflammatory Pathway	
4	Left cervical vagus implant	SetPoint Medical Corporation	RESET-RA study Climical Trial No. NCT04539964	Moderate to severe rheumatoid arthritis	
5	Spectra WaveWriter^TM^ Spinal Cord Stimulator System (VERITAS)	Boston Scientific Corporation	Clinical Trial No. NCT03251937	Chronic Pain Back Pain	
6	Precision Spinal Cord Stimulator (SCS) System (WHISPER)	Boston Scientific Corporation	Clinical Trial No. NCT02314000	Chronic Pain	
7	GiMer Medical MN 1000 External Stimulator	GiMer Medical	Clinical Trial No. NCT03285113	Failed Back Surgery Syndrome Complex Regional Pain Syndrome (CRPS)	

Bioelectronics is rapidly evolving toward the development of devices that are closed loop systems and smart systems, other than the open loop systems discussed in this review. The closed loop system requires the use of sensing technology (a technology already being used in pacemakers for the treatment of cardiac disorders) wherein parameters can be altered in response to prevailing positive and negative feedback signals. The closed loop system could be advantageous in DBS against movement disorders or in SCS in the management of pain (De Ridder et al., 2021). Smart systems, where the device is equipped with artificial intelligence (AI) which would enable it to predict the neural output in response to stimuli in its surroundings, are popular in the development of novel BEMs. An intelligent device could be used to gather and store information about the neural activity of a patient, which would enable a caregiver to customize treatment for the patient. The much sought-after application of AI in neuromodulation as of today is target localization in DBS and detection of pathological activity. There is a need to develop devices that employ AI for studying neural activity in the CNS or PNS (De Ridder et al., 2021).

Bioelectronic medicines will also aid the knowledge of the CNS and PNS with respect to coordination of neural activities and help demystify electric signaling, which controls the overall homeostasis of an individual. The information gathered could predict the dysfunction of electric signaling in an individual at an early stage and prevent the occurrence of a full-blown disorder especially with respect to incurable progressive neural degenerative diseases such as AD and PD. For many diseases that are refractory to pharmacological treatment, such as major depressive disorder, BEMs that can deliver electrical impulses through transcranial magnetic stimulation and DBS remain the only possible solution (Alliance for Advancing Bioelectronic Medicine, 2020).

## Conclusion

Recent developments in the field of science and technology have led to an emergence of innovative fabricated BEDs in the market and their applications in various health conditions are being investigated. The latest BEDs are effectively used in various fields such as diagnosis, monitoring and treatment of diseases, and/or disorders. This has also curbed the adverse effects of conventional treatments with reduction in chronic pain and risk ratio of death by enhancing quality of life. Further collaborative studies are required from experts in areas of biomedicines and engineering to bring about future innovations in BEDs and radicalize healthcare systems.

## Author Contributions

IE: visualization and writing of original draft. AU made substantial contributions to the design of the study; acquisition, analysis, and interpretation of data for study work; and drafted and revised the study for important intellectual content. SS: conceptualization, resources, review, editing, and supervision. All authors contributed to the article and approved the submitted version.

## Conflict of Interest

The authors declare that the research was conducted in the absence of any commercial or financial relationships that could be construed as a potential conflict of interest.

## Publisher’s Note

All claims expressed in this article are solely those of the authors and do not necessarily represent those of their affiliated organizations, or those of the publisher, the editors and the reviewers. Any product that may be evaluated in this article, or claim that may be made by its manufacturer, is not guaranteed or endorsed by the publisher.
